# Retraction: Activated carbons from winemaking biowastes for electrochemical double-layer capacitors

**DOI:** 10.3389/fchem.2023.1176163

**Published:** 2023-03-06

**Authors:** Frontiers Editorial Office

The Publisher retracts the 14 August 2020 article cited above.

Following publication, concerns were raised regarding the ownership and authorization of some of the data used in the study. Specifically, during the investigation, which was conducted in accordance with Frontiers’ policies, the authors failed to provide authorization for the use of the nitrogen adsorption isotherm test data. The unauthorized data use was confirmed by an investigation led by the Ethics Committee of the CSIC (Consejo Superior de Investigatcciones Cientíicas). This is a breach of Frontiers’ guidelines and those of the Committee on Publication Ethics. As such, this article is being retracted.

The authors do not agree to correspondence regarding this retraction.

This retraction was approved by the Chief Editors of Frontiers in Chemistry and the Chief Executive Editor of Frontiers.

